# Safety and tolerability of experimental hookworm infection in humans with metabolic disease: study protocol for a phase 1b randomised controlled clinical trial

**DOI:** 10.1186/s12902-019-0461-5

**Published:** 2019-12-11

**Authors:** Doris Pierce, Lea Merone, Chris Lewis, Tony Rahman, John Croese, Alex Loukas, Malcolm McDonald, Paul Giacomin, Robyn McDermott

**Affiliations:** 1 Centre for Molecular Therapeutics, Australian Institute of Tropical Health & Medicine, James Cook University, Building E5, Cairns Campus, 14-88 McGregor Rd Smithfield, Cairns, QLD 4878 Australia; 2 Centre for Chronic Disease Prevention, Australian Institute of Tropical Health & Medicine, James Cook University, QLD, Cairns, Australia; 3 The Prince Charles Hospital, QLD, Brisbane, Australia

**Keywords:** Type 2 diabetes mellitus, Metabolic syndrome, Obesity, *Necator americanus*, Human microbiota, Type 2 immune response

## Abstract

**Background:**

Abdominal obesity and presence of the metabolic syndrome (MetS) significantly increase the risk of developing diseases such as Type 2 diabetes mellitus (T2DM) with escalating emergence of MetS and T2DM constituting a significant public health crisis worldwide. Lower prevalence of inflammatory and metabolic diseases such as T2DM in countries with higher incidences of helminth infections suggested a potential role for these parasites in the prevention and management of certain diseases. Recent studies confirmed the potential protective nature of helminth infection against MetS and T2DM via immunomodulation or, potentially, alteration of the intestinal microbiota. This Phase 1b safety and tolerability trial aims to assess the effect of inoculation with helminths on physical and metabolic parameters, immune responses, and the microbiome in otherwise healthy women and men.

**Methods:**

Participants eligible for inclusion are adults aged 18–50 with central obesity and a minimum of one additional feature of MetS recruited from the local community with a recruitment target of 54. In a randomised, double-blind, placebo-controlled design, three groups will receive either 20 or 40 stage three larvae of the human hookworm *Necator americanus* or a placebo. Eligible participants will provide blood and faecal samples at their baseline and 6-monthly assessment visits for a total of 24 months with an optional extension to 36 months. During each scheduled visit, participants will also undergo a full physical examination and complete diet (PREDIMED), physical activity, and patient health (PHQ-9) questionnaires. Outcome measurements include tolerability and safety of infection with *Necator americanus*, changes in metabolic and immunological parameters, and changes in the composition of the faecal microbiome.

**Discussion:**

Rising cost of healthcare associated with obesity-induced metabolic diseases urgently calls for new approaches in disease prevention. Findings from this trial will provide valuable information regarding the potential mechanisms by which hookworms, potentially via alterations in the microbiota, may positively influence metabolic health.

**Trial registration:**

The protocol was registered on ANZCTR.org.au on 05 June 2017 with identifier ACTRN12617000818336.

Alternatively, a Google search using the above trial registration number will yield a direct link to the trial protocol within the ANZCTR website.

## Background

Since 1980, numbers of obese or overweight adults and children have grown exponentially worldwide to a current estimate of 2.1 billion people, thus establishing obesity as a pandemic and the fourth leading cause of death worldwide in 2017 [1]. Specifically, abdominal obesity has been highlighted as the central factor in metabolic syndrome (MetS), a cluster of physiological, metabolic, and biochemical risk factors including abdominal obesity, insulin resistance, hypertension, and dyslipidaemia, as it appears to precede all other factors [2]. Presence of MetS significantly increases the risk of developing several life-threatening diseases such as cancer, cardiovascular disease (CVD), and Type 2 diabetes mellitus (T2DM) [2]. With almost one quarter of the world’s population affected, the prevalence of MetS in adults in rural Australia alone is 35.8%, and numbers in the Asia-Pacific region are growing disproportionately [3], likely due to rapid socio-economic development and adoption of the Western diet and sedentary lifestyle [4]. Associated with MetS, the escalating emergence of non-communicable diseases such as CVD and T2DM constitutes a significant public health crisis for the affected regions [3].

Central and visceral obesity observed in MetS are a strong predictor for the development of T2DM, as they promote chronic inflammation and subsequent insulin resistance [5]. Worldwide numbers of patients with T2DM are on the rise, and currently 8.8% of adults are living with the disease, equating to 425 million people (http://www.diabetesatlas.org). Although the causative mechanisms are not exhaustively understood, chronic inflammation is increasingly accepted as strongly implicated in the pathogenesis of T2DM as excess calorie intake and sedentary behaviour are in the development of obesity [6]. Vascular dysfunction resulting from chronic inflammation enhances the risk of diabetic complications such as myocardial infarction, peripheral arterial disease, and diabetic nephropathy and retinopathy [7].

Interestingly, increasing incidence and prevalence of inflammatory diseases such as T2DM, inflammatory bowel disease, and asthma in developed countries offset the successful reduction in parasite-related diseases following helminth eradication efforts [8]. In 1989, the lower prevalence of inflammatory diseases in transitioning or low-middle income countries with higher incidences of helminth infections [9] led to development of the “Hygiene Hypothesis” and generated suggestions of a potential role for symbiotic parasites in the prevention and management of certain inflammatory diseases [10]. Data from cross-sectional and animal studies further corroborate this inverse relationship between helminths and inflammatory diseases. A cross-sectional study in Northern Australia unearthed an inverse relationship between infection with *Strongyloides stercoralis* and T2DM, indicating a possible protective relationship [11]. A recent meta-analysis also confirmed a potential protective nature of helminth infection against T2DM [10] with another study indicating an inverse relationship between helminth infection and CVD risk factors such as dyslipidaemia [10]. Further supporting this notion, a recent study in a helminth-endemic region of Indonesia showed that anthelminthic drug treatment of people with an active helminth infection significantly raised HOMA-IR values and insulin resistance, suggesting that the removal of worms was causally associated with worsened glycaemic control [12]. Findings from animal studies confirm the suggestion of a possible protective effect of helminth infections against T2DM and MetS via processes of immunomodulation [9], where helminth infections may dampen pro-inflammatory immune responses and skew towards a type 2 immune response [9]. A further study also demonstrated that infection with doses of 20 human hookworm *Necator americanus* (*Na*) is safe, well tolerated, and associated with Th2 and concomitant regulatory T cell responses [13, 14], identifying *Na* as a potentially effective treatment for inflammation associated with MetS and T2DM. Additionally, helminth infections may also modulate immune responses indirectly via their effect on the composition of the intestinal microbiota [15]. For example, infection of mice with gastrointestinal helminths was able to prevent colonisation with the pro-inflammatory bacterium *Bacteroides vulgatus* by inducing a Th2 and regulatory immune response that promoted the expansion of protective *Clostridiales* strains [16]. Finally, a recent review of helminths and T2DM concluded that chronic helminth infections influence the development of metabolic diseases and that these effects can be long lasting; however, human studies to date have been cross-sectional and cannot infer causality [17]. The authors called for controlled human helminth infection trials to better understand how these can modulate the immune and metabolic response.

Herein we describe a randomised, double-blind, placebo-controlled Phase 1b safety and tolerability trial that will assess the effect of inoculation with 20 or 40 infective stage three larvae (L3) of *Na* on body fat composition, inflammation and immune response, in otherwise healthy women and men aged 18–50 with central obesity and features of MetS over 24 months (Table 1).

**Table 1:** World Health Organisation Trial Registration Data Set

Data category	Information
Primary registry and trial identifying number	anzctr.org.au (ACTRN1261700818336)
Date of registration in primary registry	05/06/2017
Secondary identifying numbers	N/A
Source(s) of monetary or material support	Far North Queensland Hospital Foundation, Australian Institue of Tropical Health and Medicine
Primary sponsor	James Cook University
Secondary sponsor(s)	None
Contact for public queries	Prof Robyn McDermott robyn.mcdermott@jcu.edu.au
Contact for scientific queries	Prof Robyn McDermott, Australian Institute of Tropical Health and Medicine, Cairns, Australia
Public title	Safety and tolerability of experimental hookworm infection in humans with metabolic disease
Scientific title	Safety and tolerability of experimental hookworm infection in humans with metabolic disease: Proof of Concept (Phase 1b) clinical trial
Countries of recruitment	Australia
Health condition(s) or problem(s) studied	Diabetes, Infection
Intervention(s)	Active comparator: 20 or 40 larvae of the human hookworm *Necator americanus* (20 L3, 40 L3) over 24 months.
	Placebo comparator: Tabasco sauce solution
Key inclusion and exclusion criteria	Ages eligible for study: 18–50 years; Sexes eligible for study: both; Accepts healthy volunteers: no
	Inclusion criteria: Healthy adults 18–50 years, with central obesity (WC > 90 cm for females and > 102 cm for males) and increased insulin resistance as assessed via abnormal homeostatic model assessment of insulin resistance (HOMA-IR), i.e. HOMA-IR > 2.12 or at least two other features of MetS: elevated blood pressure > 135/85 mmHg, dyslipidaemia, or abnormal liver function test suggesting fatty liver disease. Have provided written informed consent and are willing to comply with all Protocol scheduled visits. If of childbearing potential, must be willing to use the acceptable methods of contraception.
	Exclusion criteria: Pregnancy, established chronic disease (CVD, diabetes, cancer, renal, gut disorder), history of substance abuse or current substance abuse, major allergies, known immunodeficiency disorder, asthma, taking prescribed medications or nutritional supplements likely to interfere with study outcomes, inability to provide informed consent.
Study type	Interventional
	Allocation: block randomised; intervention model: parallel assignment; Masking: double-blind, sealed envelopes and containers
	Primary purpose: prevention
	Phase 1
Date of first enrolment	March 2018
Target sample size	45
Recruitment status	Recruiting
Primary outcome(s)	Safety of experimental inoculation with 20 L3, defined by (a) Number of reported adverse events (AEs), relative to placebo cohort, (b) Assessment of general health and (c) Successful completion of 24-month trial. Adverse reactions: including but not limited to abdominal pain, rash, fever, weight loss, fatigue, nausea (mild, moderate or severe as assessed by trial doctor).
Key secondary outcome(s)	Changes in insulin sensitivity from blood pathology taken at each participant contact point during the 24-month trial; Change in BMI measured by any alteration in weight (kg) and height (cm); Change in waist circumference (cm) Change in bacterial richness of microbiome measure by shotgun assay of faecal sample

Hypotheses are that experimental infection with the human hookworm *Na*

1. will be well-tolerated and safe in this cohort of otherwise healthy but obese women and men.
2. will induce a biased Type 2 and regulatory immune response with concomitant suppression of systemic, pro-inflammatory Type 1 responses.
3. will stabilise or improve determinants of metabolic disease.
4. will modify the composition of the gut microbiota.

## Methods/design

### Study design

#### Aims

The aims of this study are

1. to establish the acceptability, safety, and tolerability of experimental infection with *Na* in obese women and men with features of MetS
2. to conduct exploratory studies into the impact of hookworms on stabilising or improving metabolic, immune/inflammatory, and microbiological parameters associated with metabolic disease

#### Design

**Fig. 1:**
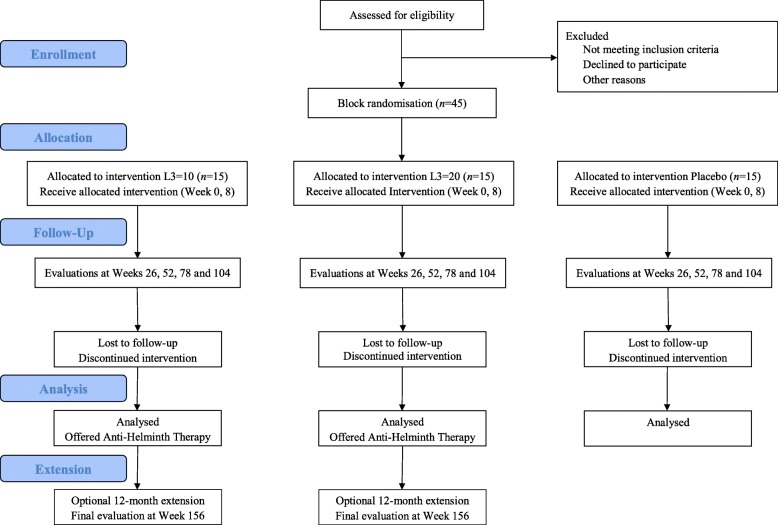
Trial design flowchart

This study is designed as a randomised, double-blind, placebo-controlled Phase 1b safety and tolerability trial. An initial recruitment target of 54 allows for a drop-out rate of 20%, leaving 45 volunteers to participate in the trial. Volunteers who meet the eligibility criteria, have been approved following screening, and have given informed consent (Additional file 1) will be randomised into three trial groups. Groups 1 and 2 will be inoculated with either 20 (2 × 10, *n* = 15) or 40 (2 × 20, *n* = 15) L3 of *Na* with Group 3 (Placebo, *n* = 15) administered Tabasco® sauce in a solution identical in appearance to the L3 solutions. All groups will be monitored over a period of at least 24 months followed by an optional extension to 36 months to assess the impact of eliminating the worm infection following anti-worm medication (Fig. 1).

#### Setting

Participants will be recruited from the Cairns and Townsville regions in Far North Queensland, Australia. As of June 2016, the regions' estimated populations were 162,000 (www.cairns.qld.gov.au Accessed 15 November 2018) and > 185,000, respectively (http://www.population.net.au/townsville-population Accessed 3 December 2019). The study site is the James Cook University campus, located in Cairns and Townsville. The human hookworm *Na* is not endemic to these metropolitan areas.

### Participant characteristics

Participant recruitment initiatives include advertising in local media, notices around Cairns including the James Cook University (JCU) Cairns campus, student and staff email lists (with permission from the university), and social media.

#### Inclusion criteria

**Fig. 2:**
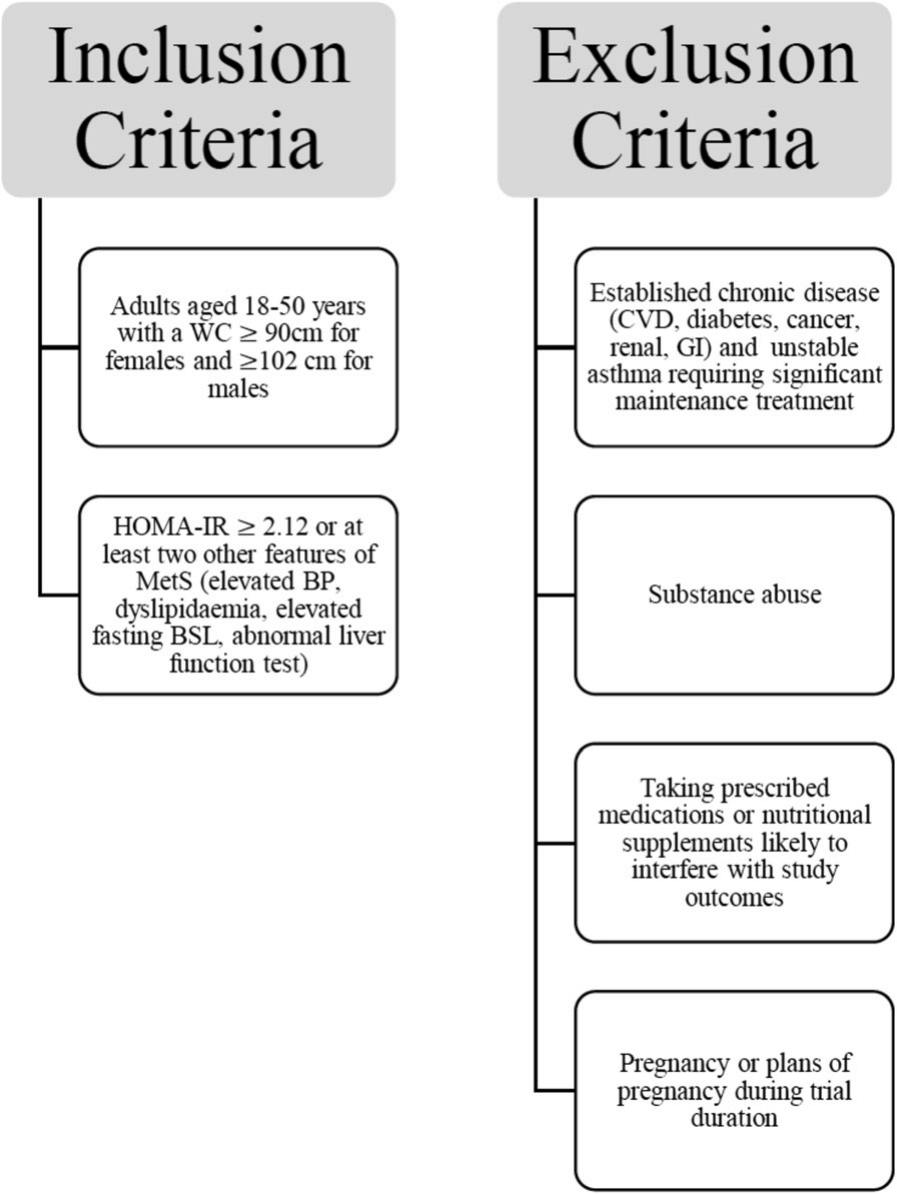
Flow diagram of inclusion/exclusion criteria. WC, waist circumference; CVD, cardiovascular disease; GI, gastro-intestinal; HOMA-IR, homeostatic model assessment of insulin resistance; MetS, metabolic syndrome; BP, blood pressure; BSL, blood sugar level

Eligible for inclusion into this study are otherwise healthy women and men in the primary age window for progression to T2DM (18–50 years) [18] with central obesity (waist circumference, WC > 90 cm for women and > 102 cm for men) and increased insulin resistance as assessed via abnormal homeostatic model assessment of insulin resistance (HOMA-IR), i.e. HOMA-IR > 2.12 [19] or at least two other features of MetS: elevated blood pressure (> 135/85 mmHg), dyslipidaemia (elevated total cholesterol triglycerides, LDL or total cholesterol/HDL ratio, low HDL), elevated fasting blood sugar level, or abnormal liver function tests (abnormal alanine transaminase, aspartate transaminase, alkaline phosphatase, albumin and total protein, globulin, bilirubin, and gamma-glutamyl transferase) suggesting fatty liver disease as per pathology report (Fig. 2, Additional file 2). Volunteers must be willing to comply with all protocol scheduled visits and provide written informed consent prior to study entry with the option to withdraw at any time. Women of child bearing potential should agree to use acceptable methods of contraception.

#### Exclusion criteria

Exclusion criteria include pregnancy, established chronic disease (CVD, diabetes, cancer, renal, gastrointestinal disorders), history of substance abuse, current substance abuse, major allergies, known immunodeficiency disorder, unstable asthma requiring significant maintenance treatment, taking oral prescription medications or nutritional supplements likely to interfere with study outcomes, and inability to give informed consent (Fig. 2).

### Processes, interventions, and comparisons

#### Initial clinical assessment and sample collection/week − 6

During the first visit, consenting participants will be interviewed and examined by the trial doctor and eligibility determined based upon inclusion criteria, medical history, pre-existing conditions, current medications as well as recent travel to worm-endemic countries, known previous worm infections, and deworming treatments. The trial doctor will also conduct a full physical examination during initial screening including height, weight, examination of respiratory, cardiovascular and nervous systems, and a pregnancy test for females. Participants will be asked to attend Sullivan Nicolaides Pathology (SNP) for a fasting blood test assessing lipid profile, liver function, fasting glucose and insulin (Table 2).

**Table 2:**
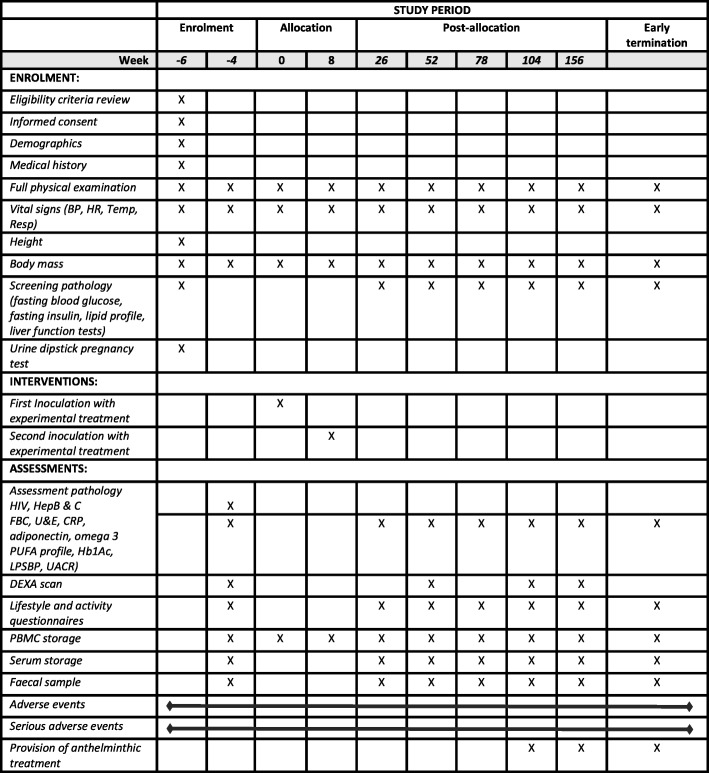
Schedule of investigations

BP, blood pressure; HR, heart rate; Temp, temperature; Resp, respiratory rate; HIV, human immunodeficiency virus; HepB & C, hepatitis B and hepatitis C; FBC, full blood count; U & E, urea and electrolytes; CRP, C-reactive protein; PUFA, polyunsaturated fatty acids; Hb1Ac, glycated haemoglobin; LPSBP, lipopolysaccharide-binding protein; UACR, urinary albumin-creatinine ratio; DEXA, dual-energy X-ray absorptiometry; PBMC, peripheral blood mononuclear cells

The study team will contact participants after the screening visit to inform them of their eligibility to proceed. If eligible, the study team will book the next visit according to the study schedule. Clinical assessments will consequently be completed during all follow-up assessments to ensure patient safety and monitoring. Pathology sampling (blood and faeces) will be repeated at intervals throughout follow-up. The schedule of visit requirements is detailed below.

#### Baseline visit/week − 4

During this visit, the study doctor will review the laboratory test results, confirm continued eligibility for participation, perform a full physical examination, and administer diet, health and activity questionnaires, which have been previously validated [20–23]. Blood and faecal samples will be collected and body composition determined via dual-energy X-ray absorptiometry (DEXA) (Table 1). The study doctor will obtain written informed consent for storage of health information and/or blood or tissue samples in the university’s Biobank.

#### Treatment period/week 0 to week 104

The treatment period will last up to 104 weeks during which participants will be required to attend the clinic for two inoculation visits eight weeks apart and four evaluation visits approximately 26 weeks apart. Participants may also have telephone and/or Skype appointments with a designated study team member to monitor their health status and promote retention; these visits will be scheduled with participants by negotiation. At the end of the treatment period, participants will have their final blood and stool tests and DEXA scan, complete the scheduled questionnaires, and undertake an exit consultation and examination with the study doctor. Furthermore, as a mandatory requirement of the study, participants will be provided with the anthelmintic treatment mebendazole (100 mg to be taken twice daily for 3 days to clear the hookworm infection) at no cost to them; however, it is not mandatory for participants to take this medication. Participants electing to refrain from taking the anthelmintic treatment (that is choosing to keep their helminths) following completion of the 104-week period will be advised that their future medical care will be passed onto their general practitioner. At 104 weeks, the study will become unblinded, and there will be an optional 52-week extension to the study for willing participants to examine the impact of de-worming medication on safety, immunological and metabolic parameters.

##### Randomisation and inoculation (week 0 and week 8)

Previous studies have established the safety of experimental inoculation with *Na* in dose-controlled conditions [14, 24] and indicate that *Na* is well tolerated in human trial subjects, up to a dose of 25 third stage larvae (L3), beyond which adverse effects such as abdominal pain become more problematic [25]. Doses as high as 50 L3 have been associated with abdominal pain, nausea and oedema [26]; therefore, doses of 20 and 40 L3 have been selected for the current study and are consistent with doses from a parallel study in people with coeliac disease (Trial identifier NCT02754609). The allocation sequence for the three cohorts was generated by random block number generation (block size of 6). The unblinded researcher will subsequently implement allocation using sequentially numbered, opaque, sealed envelopes and containers. The inoculum, either 10 x L3, 20 x L3 or Tabasco (placebo) will be prepared by the unblinded research assistant freshly on site following an established protocol that has been used for similar previous studies [14]. Larvae are decontaminated using iodine disinfectant and individually selected by an experienced researcher based on morphological integrity and active motility, so we can be certain that larval viability is optimal. All inocula will appear identical, and study doctor, investigators, participants, and data analysts will be blinded. Consenting participants, depending on randomised group allocation, will be inoculated with *Na* larvae, either 10 x L3 or 20 x L3 in 300 μL of de-ionised water. Two doses will be administered 8 weeks apart (Table 2) by applying the solution to a dressing pad, which will be placed on the participant’s forearm. Participants will be advised that there may be localised itch and irritation at the inoculation site, much the same as would be experienced if chilli pepper were to be rubbed into the skin. Following inoculation, participants will be observed for one hour in case of an adverse response. One medical monitor will remain un-blinded for the study duration. Unblinding of participants may occur upon advice from the safety management committee in case of a serious adverse effect (SAE). Participants will be instructed of the importance of maintaining their normal hygiene practices (using a toilet) during the study. This is essential for preventing parasite eggs that are shed in the faeces from developing in the environment into the infective stage larvae, a process that takes 7–10 days.

##### Safety and clinical monitoring

During the initial screening visit and every visit thereafter (or as required in the event of an adverse event), general health assessments, physical examination and vital signs (blood pressure, heart rate, temperature and respiratory rate) will be obtained to ensure safety and monitoring. Urine pregnancy test will be performed for female participants at the beginning of the study (positive tests exclude the participant from the trial) and urinary beta human chorionic gonadotropin testing will be performed during the study, if pregnancy is suspected. Should pregnancy occur during the study, the participant will be treated as an Early Termination.

Incidence and severity of adverse events (AEs) and SAEs will be evaluated formally via structured questionnaires (Additional file 3) reviewed by the investigating clinician and informally via participant-initiated contact. The most significant side effects are likely to occur during the first 4–6 weeks following inoculation as the worms anchor themselves to the intestinal mucosa to facilitate feeding and avoid ejection by gut peristalsis [27]. Common side effects during this initial colonisation include abdominal pain, increased flatulence, nausea, and bloating [28]. The second inoculation visit eight weeks after the first inoculation will provide an ideal opportunity to identify/monitor any side effects. Potential side effects have also been outlined in an extensive participant information sheet provided prior to informed consent and are reiterated during the first inoculation visit. Participants are encouraged during the first inoculation visit to contact the trial doctor should they notice any unusual signs and symptoms. If deemed necessary by the trial doctor, participants will be called in for an additional examination, during which they can elect to discontinue with the trial. Should the symptoms be consistent with potential side effects of hookworm infection, the participant may become unblinded and offered anthelmintic medication.

Pathology samples (blood) will be monitored every 6 months for incidence and severity of laboratory abnormalities (Table 2). In the event of a SAE related to the trial, the participant will firstly be referred to their own GP and the case referred to an independent safety committee. With the consent of the participant, their regular GP will be notified routinely of their participation in the study at commencement. This will be in the form of a letter stating the nature of the study, objectives, and concomitant drugs to be avoided; the participant will also receive a copy of this letter.

#### Outcomes

The primary outcome measure will be the safety of experimental hookworm infection of otherwise healthy women and men with 20 *Na* L3 as evaluated by number of reported AEs and SAEs, assessment of general health, and successful completion of the 24-month trial.

Secondary outcomes include:

1. safety/tolerability of the higher dose of 40 *Na* L3
2. longitudinal and inter-cohort changes in metabolic parameters (HOMA-IR, DEXA, WC, body mass index, fasting insulin and glucose, lipopolysaccharide-binding protein, glycated haemoglobin, blood pressure, resting heart rate, C-reactive protein, adiponectin, full blood count, white blood cell count, omega-3 poly-unsaturated fatty acid profile, urinary albumin-creatinine ratio)
3. immunological parameters (fluctuations in blood and serum Type 1 and Type 2 immune responses)
4. longitudinal and inter-cohort changes in the composition of the faecal microbiome
5. impact of de-worming medication on, safety, immunological and metabolic markers

### Data collection, management, and statistical analyses

All eligible participants will be assigned a unique identification number at their first screening visit. Participant confidential data, including anthropometric measures, lifestyle, and medical history, will be recorded on a hardcopy Case Report Form (Additional file 4), which will then be entered into a password protected electronic database developed specifically for this trial. Hard copy Case Report Forms will be stored in participant trial folders in a locked cabinet in a secure environment at the JCU Cairns campus. Access to the folder containing the databases will be restricted to study personnel only, who will have signed a confidentiality agreement as a condition of their involvement in the study. Data from the final trial data set will be provided to investigators and statisticians in de-identified electronic format and this data will be linked to Case Report Form data using the unique identification number, initials, and date of collection. While a data management team was not formed due to minimal risks associated with the study, data will be audited regularly by independent monitors for independent source data verification.

At trial conclusion, each participant will be provided with an individual report including their baseline test results and any changes over the study period. At this time, they will also be informed of study allocation (20 x L3, 40 x L3, placebo). Upon publication of results, the participants can opt to receive a copy of the manuscript.

#### Sample size

This Phase 1b safety and dose-ranging study will include 45 participants with 15 to be infected with 20 x L3, 15 to be infected with 40 x L3, and 15 in the placebo control group. In the absence of other effect size data from human trials upon which to base a power calculation, and as a change in HOMA-IR from Baseline is a primary metabolic outcome of this study, we have adapted the SUGARSPIN HOMA-IR result [29] and assume an effect size of 1.06 over 2 years. A total of 15 participants in each group reflects 80% power to detect an effect size of 1.06 using the T-statistic and 1.023 using Z-statistic. The aim to recruit 54 participants allows for a potential drop-out rate of 20%.

#### Data processing and statistical analyses

##### Safety of infection

The primary outcome, the safety of infection with 20 *Na* L3, will be assessed using a chi-squared test of proportions to determine any differences in the proportions satisfying the safety criteria at 24 months between the placebo, 20 x L3 and 40 x L3 groups. Kaplan Meir models will be used to assess progression throughout the study, and Cox regression models will assess safe progression through the study. Standard descriptive statistics will be performed on all outcome measures at Baseline, all interim points of assessment, and at 24 months after inoculation (categorical variables: absolute and relative frequencies; numerical variables: mean and standard deviation or median and interquartile range, dependent on data distribution). Successful *Na* infection will be confirmed using qPCR detection of parasite eggs in faecal samples taken at Week 26. The faecal sample collected at Baseline (Week -4) will be used for assessment of current or previous helminth infections that could have interfered with trial outcomes.

##### Metabolic parameters

Changes in metabolic determinants, in particular changes in body mass, visceral fat mass (DEXA), insulin sensitivity (HOMA-IR), and adiponectin will be analysed via comparison of Baseline and follow-up data and visually checking the outcome measures moved in the expected direction for each allocation group. Paired statistical analysis (Chi-square and t-tests) may be conducted between intervention and control groups, if the sample size warrants.

##### Impact of helminth infection on immune status

Serum samples collected at each scheduled visit will be batch-analysed using a custom-panel of 17 cytokines (IFNγ, IL-1β, IL-12p70, IL-2, IL-4, IL-6, TNF, IL-10, IL-17A, IL-12p40, IL-5, IL-13, IL-15, IL-18, IL-33, MCP-1, MIP-1α) for circulating inflammatory biomarkers associated with metabolic disease using cytometric bead array. Mean cytokine levels in placebo and hookworm groups will be compared by general linear models. Peripheral blood mononuclear cells will be processed at scheduled visits and analysed using flow cytometry for expression of markers of cell phenotype (CD3, CD19, CD4, CD8), activation (CD27, CD45RA, CD45RO, CD69) and function (CD25, CRTH2, CTLA4, FOXP3, IL-2, IL-4, IL-5, IL-10, IL-17A, IFNγ and TNF).

##### Impact of helminth infection on faecal microbiota

Faecal specimens collected in anaerobic sample collection containers will be stored at − 80 °C and, later, DNA will be extracted prior to genome-wide shotgun sequencing to describe the composition and metabolic potential of the microbiota. Following quality filtering, the QIIME pipeline will be used for operational taxonomic unit (OTU) analysis and Beta diversity will be analysed using UniFrac. Longitudinal comparisons will be made between Baseline, 12 and 24 months looking at diversity and any changes in OTUs between treatment and placebo groups. These data, along with clinical and immunological data, will be analysed subsequently in an integrative fashion to more comprehensively define the biological impact of experimental *Na* infection on individuals with metabolic disease.

##### Missing data and early termination

Two research team members will review data from each participant to identify and address missing information. To account for missing data at the analysis stage, this study will follow the method prescribed in Jakobsen et al. [30]. Specifically, complete case analysis will be used as the primary analysis if the proportion of missing data is considered negligible (i.e. < 5%) and the number of participants lost to follow up is comparable across study groups. Multiple imputation will be trialled during analysis should missing data exceed 5%, is spread across both independent and dependent variables, and is likely missing at random. Should missing data exceed 40%, no imputation will be undertaken and results will be interpreted in the context of this limitation [30]. Early termination and withdrawals from the trial will be recorded throughout the intervention and evaluation periods.

## Discussion

To our knowledge, this is the first study to examine the long-term safety/tolerability of experimental inoculation with different doses of the hookworm *Na* in human subjects with central adiposity and signs of MetS. With safety of helminth therapy as the primary outcome, this study will also allow observation and quantification of the longer-term effects of helminth therapy on metabolic disease progression, providing a foundation for future research into dose-controlled helminth-based therapies.

The steeply rising cost of healthcare associated with overweight and obesity-induced metabolic diseases, including T2DM, in Australia [31] and increasing cases of obesity and T2DM in children of obese or overweight mothers [32] urgently calls for new approaches in preventing disease progression. Several compelling lines of evidence from animal models and human studies point to a potential role for helminths in significantly reducing the impact of obesity on disease progression. Pathways may involve skewing the immune response to a Th2 or regulatory (anti-inflammatory) response as well as alteration of the gut microbiota with helminth infection. We are pioneering the safe delivery of experimental *Na* infection in human trials and are, thus, uniquely placed to undertake a world-first proof-of-principle trial to determine the safety and effectiveness of *Na* infection in limiting the inflammatory and metabolic cascades that are associated with obesity and T2DM.

Recent research has highlighted the complex interplay between the organisms that comprise our intestinal microbiota (commensal bacteria), macrobiota (helminths), and the host mucosal immune response. Consequently, the absence of either micro- or macrobiota may disturb homeostasis in the host and predispose the immune system to a more pro-inflammatory response [33]. Helminths can directly influence the immune regulatory function via secretion of various products and indirectly via alteration of the microbiota [33]. In turn, alteration of the microbiota can also promote or depress helminth presence in the host [33]. Critically, these interactions are fundamental in shaping the health and homeostasis of the immune system; however, the mechanisms involved remain poorly understood. The present trial will offer a rare opportunity to examine the nature, kinetics, and interactions of parasite-induced changes in systemic immune responses, in composition of the gut microbiota, and in metabolic status within the carefully controlled context of a hookworm challenge trial. The findings will provide valuable information regarding the potential mechanisms by which hookworms, potentially via alterations in the microbiota, may positively influence metabolic health.

While live worm therapy is unlikely to be a widely acceptable therapy in T2DM prevention for the general public, discovering novel immune-regulatory, worm-secreted molecules may attract investment from pharma for other inflammatory conditions. Hence, this trial will be fundamental for providing the rationale for future endeavours to identify hookworm-secreted factors that could be tested as therapeutics that are more amenable to wider populations and have commercial potential.

## Supplementary information

**Additional file 1.** Participant Consent form: The Informed Consent form used in the present clinical trial.

**Additional file 2.** Screening pathology form: The Laboratory Request form used for to assess eligibility of participants following their initial screening visit.

**Additional file 3.** Adverse event Case Report Form: The Case Report form used to record Adverse Events.

**Additional file 4.** Screening Case Report Form: The Case Report form used during the initial Screening visit to assess eligibility for the current trial.

## Data Availability

Data sharing is not applicable to this article, as no datasets were generated or analysed for this study protocol.

## Abbreviations

AE: Adverse event
CVD: Cardiovascular disease
DEXA: Dual-energy X-ray absorptiometry
HOMA-IR: Homeostatic model assessment of insulin resistance
L3: Third stage larvae
MetS: Metabolic syndrome
*Na*: *Necator americanus*
OTUs: Operational taxonomic units
SAE: Significant adverse event
T2DM: Type 2 diabetes mellitus
Th2: T helper type 2
WC: Waist circumference
