# Supplementary material for: Safety and tolerability of experimental hookworm infection in humans with metabolic disease: study protocol for a phase 1b randomised controlled clinical trial
Source: BMC Endocr Disord. 2019 Dec 11;19:136. doi: 10.1186/s12902-019-0461-5 (PMC6907345; doi:10.1186/s12902-019-0461-5)
Supplement: Supplementary file 1 — Additional file 1. Participant Consent form: The Informed Consent form used in the present clinical trial. [file 12902_2019_461_MOESM1_ESM.pdf]

INFORMED CONSENT FORM v3.0 30/8/17

PRINCIPAL INVESTIGATOR: Prof Robyn McDermott
PROJECT TITLE: Safety and tolerability of experimental hookworm infection in humans with metabolic disease: a double blind randomised controlled phase 1 trial”
COLLEGE: Public Health, Medical and Veterinary Sciences

I understand the aim of this research study is to determine the tolerability and effectiveness of experimental hookworm infection in potentially preventing diabetes and metabolic syndrome in otherwise healthy overweight young women at high risk of type 2 diabetes. I consent to participate in this project, the details of which have been explained to me, and I have been provided with a written information sheet to keep.

I understand that my participation will involve a series of ***complete physical medical examinations, completion of health and lifestyle questionnaires, completion of a food diary, pathological testing of blood, faeces and urine samples, body composition imaging and potential inoculation with experimental hookworm over a three year period*** and I agree that the researcher may use the results as described in the information sheet.

I acknowledge that:

- any risks and possible effects of participating in the “***Safety and tolerability of experimental hookworm infection in humans with metabolic disease: a double blind randomised controlled phase 1 trial”*** have been explained to my satisfaction;
- taking part in this study is voluntary and I am aware that I can stop taking part in it at any time without explanation or prejudice and to withdraw any unprocessed data I have provided;
- that any information I give will be kept strictly confidential and that no names will be used to identify me with this study without my approval;

(Please tick to indicate consent)

**I consent to take part in the proposed research study, the nature and purpose of which has been explained to me.**		**Yes**		**No**
**I consent to undergo baseline screening tests and, if deemed eligible to participate in the trial, undergo further testing and clinical visits according to the schedule in the Patient information Sheet Provided**		**Yes**		**No**
**I consent to the sharing of my information about participation in this study with my regular General Practitioner (GP)**		**Yes**		**No**
**I consent to the storing of health information and/or blood or tissue for future research studies. I understand that these samples may be retained after the completion of the initial laboratory tests, and will be destroyed when it is determined that all future research is complete**		**Yes**		**No**
**I understand that at the end of the trial I will be given effective hookworm eradication treatment**		**Yes**		**No**
**I understand that should I choose not to receive hookworm eradication treatment that responsibility for any further medical care will be transferred to my regular General Practitioner but I may be asked to attend a 1 year follow-up testing and clinical visit**		**Yes**		**No**

**Name:** *(printed)*	
**Signature:**	**Date:**
