# Supplementary material for: Safety and tolerability of experimental hookworm infection in humans with metabolic disease: study protocol for a phase 1b randomised controlled clinical trial
Source: BMC Endocr Disord. 2019 Dec 11;19:136. doi: 10.1186/s12902-019-0461-5 (PMC6907345; doi:10.1186/s12902-019-0461-5)
Supplement: Supplementary file 2 — Additional file 2. Screening pathology form: The Laboratory Request form used for to assess eligibility of participants following their initial screening visit. [file 12902_2019_461_MOESM2_ESM.pdf]

Sullivan Nicolaides PATHOLOGY
Quality is in our DNA

Clinical Trials

Laboratory Request Form

PATIENT IDENTIFICATION
SUBJECT INITIALS | SUBJECT NUMBER

SEX

DATE OF BIRTH

ADDRESS
Hookworm Infection Study, James Cook University, Cairns 4870

TESTS REQUESTED

SCREENING

Fasting Blood Glucose	FPG
Fasting Insulin	IN
CHDL *(Cholesterol, Triglyceride, HDL, LDL)*	CHDL
Liver Function Test	LFT

Is patient fasting? ☐ **Yes** ☐ **No**

Date of Collection: ☐☐ / ☐☐☐ / ☐☐☐☐
DD / MMM / YYYY

Time of Collection: ☐☐ : ☐☐
24hr clock

0 0 9

………………………………………../……/……

SULLIVAN NICOLAIDES PTY LTD ACN 078 202 196 APA *24 Hurworth Street Bowen Hills 4006*

COPY REPORTS TO
SNP Clinical Trials Manager

REFERRER
H15899
Trial Coordinator

H15899
B7052

ACCOUNT NAME
Hookworm Infection Study

COLL INIT	LOC CODE					
U	BR400	**SST** Tube	**EDTA** Tube	**LIH** Tube	**CIT** Tube	**PPT** Tube
		**ACD** Tube	**Vacu** Tube	**Rand** Urine	**Jar** Other	**Histo**
COLL CODE	PAY CAT	**Pap** Slide	**ThP** ThinPrep	**Chlam** Swab	**Trans** Red swab	**Plain** Black wab
D	**HOOKS**	**Unspun**	**Frozen**	**Card**	**Other**	

Attention Collection Staff

1. Collect **1 x 8.5ml SST Vacutainer**. Gently invert x 5.
2. Centrifuge SST.
3. Transport all specimens to SNP Bowen Hills.
