# Supplementary material for: Safety and tolerability of experimental hookworm infection in humans with metabolic disease: study protocol for a phase 1b randomised controlled clinical trial
Source: BMC Endocr Disord. 2019 Dec 11;19:136. doi: 10.1186/s12902-019-0461-5 (PMC6907345; doi:10.1186/s12902-019-0461-5)
Supplement: Supplementary file 3 — Additional file 3. Adverse event Case Report Form: The Case Report form used to record Adverse Events. [file 12902_2019_461_MOESM3_ESM.pdf]

CASE REPORT FORM<br>WAM Clinical Trial	Visit Date									
		D	D	M	M	M	Y	Y	Y	Y
Adverse Event	Trial Identifier									

**Last previous visit week (0,8,26, etc.)**	
**Adverse Event description**	____________________<br>____________________<br>____________________
**Onset date of Adverse Event**	Date / /
**Adverse Event ongoing (circle one)**	Yes / No
**End date of Adverse Event (if no longer ongoing)**	Date / /
**Severity (tick one)**	Mild ☐<br>Moderate ☐<br>Severe ☐
**AE meets Severe Adverse Event Criteria (circle one)**	Yes / No
**Event reported to ethics committee? (circle one)**	Yes / No
**Relationship to Hookworm treatment (tick one)**	Not related ☐<br>Unlikely ☐<br>Related ☐<br>Possibly related ☐<br>Definitely related ☐

Relevant Notes:

....

....

....

....

....

....
