# Supplementary material for: Safety and tolerability of experimental hookworm infection in humans with metabolic disease: study protocol for a phase 1b randomised controlled clinical trial
Source: BMC Endocr Disord. 2019 Dec 11;19:136. doi: 10.1186/s12902-019-0461-5 (PMC6907345; doi:10.1186/s12902-019-0461-5)
Supplement: Supplementary file 4 — Additional file 4. Screening Case Report Form: The Case Report form used during the initial Screening visit to assess eligibility for the current trial. [file 12902_2019_461_MOESM4_ESM.pdf]

CASE REPORT FORM WAM Clinical Trial	Visit Date									
		D	D	M	M	M	Y	Y	Y	Y
Visit 1 Week -6	Trial Identifier									

CONSENT

Date of Informed Consent: day ___ month ___ year 2 0 1 _

IMPORTANT: Informed consent must be obtained from the participant before study procedures are started.

PARTICIPANT INFORMATION:

FIRST NAME:

MIDDLE NAME:

LAST NAME:

ADDRESS:

PHONE:

EMAIL:

OCCUPATION:

PRIMARY CARE PHYSICIAN:

ADDRESS:

Date of Birth: day ___ month ___ year ___ Age (years) ___ GENDER M / F / O ___

INDIGENOUS STATUS: (Tick ONE box only)

- ☐ ABORIGINAL but not Torres Strait Islander origin
- ☐ TORRES STRAIT ISLANDER but not Aboriginal origin
- ☐ BOTH Aboriginal and Torres Strait Islander origin
- ☐ NEITHER Aboriginal or Torres Strait Islander origin
- ☐ NOT STATED / UNKNOWN / OTHER (PLEASE SPECIFY)

Waist Circumference (cm) ___

Height (cm): ___ Weight (kg): ___ BMI ___

CASE REPORT FORM<br>WAM Clinical Trial	Visit Date									
		D	D	M	M	M	Y	Y	Y	Y
Visit 1 Week -6	Trial Identifier									

PAST MEDICAL AND SURGICAL HISTORY

CURRENT MEDICATIONS:

CASE REPORT FORM WAM Clinical Trial	Visit Date									
		D	D	M	M	M	Y	Y	Y	Y
Visit 1 Week -6	Trial Identifier									

SMOKING:

	No	Yes
**Are you a current smoker?** (Tick ONE box only)<br>➢ 100 cigarettes in their lifetime and has smoked within 28 days	☐	☐
If YES, How many cigarettes per day do you smoke?		

	No	Yes
**If NO, Have you ever smoked?** (Tick ONE box only)<br>➢ 28 days duration, previously having smoked > 100 cigarettes in their lifetime	☐	☐

ALCOHOL:

	No	Yes
	☐	☐

Estimated units per week ……………………………………………………………………………………..

………

………

	No	Yes
Ever sought help for alcohol addiction?	☐	☐

ILLICIT SUBSTANCES:

	No	Yes
	☐	☐

Specify:

………

………

………

………

CASE REPORT FORM<br>WAM Clinical Trial	Visit Date									
		D	D	M	M	M	Y	Y	Y	Y
Visit 1 Week -6	Trial Identifier									

**Blood Pressure**	**1.**
	**2.**
	**3.**
	**Mean:**
**Heart Rate**	
**Temperature**	
**Respiratory Rate**	

Full Clinical Examination Findings:

……

……

……

……

……

……

……

……

……

……

……

CASE REPORT FORM<br>WAM Clinical Trial	Visit Date									
		D	D	M	M	M	Y	Y	Y	Y
Visit 1 Week -6	Trial Identifier									

	POSITIVE	NEGATIVE
Urine Pregnancy Test:		

	No	Yes
Pathology Form given:		

Trial Participation	No	Yes
**Exclude**:		

Reason for exclusion:

…………………………………………………………………………………………………….

…………………………………………………………………………………………………….

…………………………………………………………………………………………………….

…………………………………………………………………………………………………….

…………………………………………………………………………………………………….

…………………………………………………………………………………………………….

…………………………………………………………………………………………………….

…………………………………………………………………………………………………….

…………………………………………………………………………………………………….

…………………………………………………………………………………………………….
